# Are Current Lipid Guidelines Adequate for South Asian Patients? A Narrative Review

**DOI:** 10.5334/gh.1590

**Published:** 2026-10-08

**Authors:** Kavindya Fernando, Shamila De Silva

**Affiliations:** 1 Medical Research Institute, Borella, Sri Lanka; 2 NK Ceylon Institute of Medical Research, Negombo, Sri Lanka; 3 Faculty of Medicine, University of Kelaniya, Sri Lanka; 4 Colombo North Teaching Hospital, Ragama, Sri Lanka

**Keywords:** South Asian, cardiovascular risk, lipid guidelines, apolipoprotein B, lipoprotein(a), coronary artery calcium, PREVENT equations, risk stratification

## Abstract

South Asians, who account for nearly one quarter of the world’s population, experience a disproportionately high burden of atherosclerotic cardiovascular disease (ASCVD), often presenting 5–10 years earlier than European populations. This review evaluates whether contemporary lipid-management frameworks adequately address cardiovascular risk in South Asians. South Asians exhibit a distinct lipid phenotype characterised by elevated apolipoprotein B (ApoB), hypertriglyceridaemia, reduced HDL-cholesterol (HDL-C), increased small dense LDL (sdLDL) particles, and a high prevalence of elevated lipoprotein(a) (Lp(a)), features that are incompletely captured by conventional LDL-cholesterol (LDL-C)-based approaches. Existing cardiovascular risk tools show variable performance in South Asian populations, while contemporary guidelines differ substantially in risk assessment, LDL-cholesterol targets, incorporation of risk enhancers, and use of coronary artery calcium (CAC) scoring. Although recent guideline updates increasingly acknowledge South Asian ethnicity as a risk modifier, important gaps in calibration and implementation remain. Greater harmonisation of recommendations and validation of South Asian-specific risk assessment strategies are needed to improve cardiovascular prevention.

## Graphical Abstract

**Figure:**
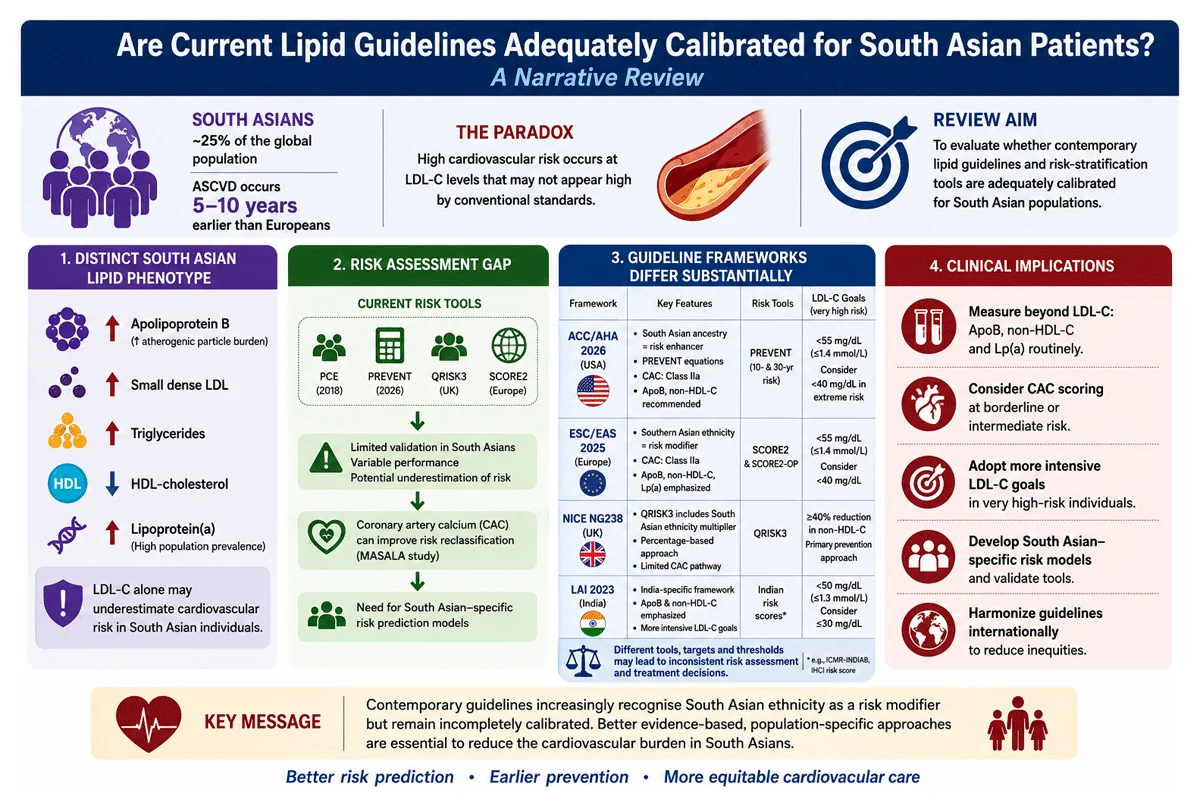


## 1. Introduction

Cardiovascular disease is the leading cause of mortality globally, yet its burden is not distributed equally. South Asians, encompassing the populations of India, Pakistan, Bangladesh, Sri Lanka, and Nepal, together with their diaspora communities across the UK, US, Canada, and Australia, comprise approximately 25% of the global population and experience atherosclerotic cardiovascular disease (ASCVD) at a rate and severity disproportionate to their conventional risk-factor profile (1,2). Myocardial infarction occurs 5–10 years earlier in South Asians than in European-origin comparators, presents more frequently without antecedent angina, and carries a higher case-fatality rate (2,3). This excess risk is not explained by the established cardiovascular risk factors captured in standard clinical assessment, nor is it adequately addressed by the guidelines those assessments are designed to inform.

The central paradox of South Asian cardiovascular risk is lipid mediated. South Asians frequently develop ASCVD at LDL-cholesterol (LDL-C) concentrations that may not appear markedly elevated by conventional risk-assessment frameworks. The explanation lies not in LDL-C itself but in a distinct atherogenic dyslipidaemia, characterised by elevated apolipoprotein B (ApoB), sdLDL particles, hypertriglyceridaemia, suppressed HDL-cholesterol (HDL-C), and a high population prevalence of elevated lipoprotein(a) (Lp(a)), for which standard lipid panels and risk calculators have shown variable calibration in South Asian populations (4,5). The clinical consequence is a population in which cardiovascular risk is routinely underestimated, treatment is inadequately initiated, and targets—once treatment has begun—are insufficiently ambitious.

Guidelines have begun to acknowledge South Asian ethnicity as a cardiovascular risk modifier. The 2026 ACC/AHA/Multisociety Dyslipidemia Guideline, which retires and replaces the 2018 guideline, explicitly names South Asian ancestry as a risk-enhancing factor and restores numerical LDL-C targets, representing the most comprehensive update to US lipid guidance in nearly a decade (6,7,8). The National Institute for Health and Care Excellence (NICE) NG238 embeds ethnicity multipliers within QRISK3, and the 2023 Lipid Association of India (LAI) Consensus Statement IV provides the first Indian-population-focused risk stratification and treatment framework, developed specifically to address the heightened cardiovascular risk and characteristic lipid phenotype observed in Indian patients (9,10). However, acknowledgement is not equivalence. No validated cardiovascular risk equation exists for native South Asian populations. The 2026 guideline’s replacement of the Pooled Cohort Equations (PCE) with the Predicting Risk of Cardiovascular Disease EVENTs (PREVENT) equations, which generate risk estimates 40–50% lower for equivalent clinical profiles, introduces a new and as-yet unquantified risk of downward misclassification in South Asian patients. Trial evidence underpinning lipid-lowering therapy derives overwhelmingly from European-origin cohorts: South Asians constituted just 3.2% of participants across 310 major cardiovascular randomised controlled trials (11). The tools available to clinicians caring for South Asian patients were not built for them.

This narrative review addresses that gap. It examines whether current cardiovascular risk-stratification tools and lipid guideline frameworks are adequately calibrated for South Asian populations in both native and diasporic settings, synthesising evidence across lipid-phenotype characterisation, risk stratification, and guideline comparison. The central question asked here is simple, and its answer has direct consequences for millions of patients: are current lipid guidelines adequate for South Asian patients?

This narrative review is based on a search of PubMed and Scopus for literature published between January 2015 and May 2026. Guideline documents, cohort studies, and trial data addressing the South Asian lipid phenotype, cardiovascular risk stratification, and guideline applicability were selectively synthesised. As a narrative rather than systematic review, article selection was based on relevance and was not governed by a prespecified protocol, formal inclusion/exclusion criteria, or structured quality appraisal.

## 2. The South Asian Lipid Phenotype

### 2.1 Atherogenic dyslipidaemia and ApoB discordance

South Asians exhibit a distinct atherogenic dyslipidaemia characterised by hypertriglyceridaemia, reduced HDL-C, elevated ApoB, and an increased proportion of sdLDL particles, a pattern that persists even when LDL-C remains within the normal range (Table 1) (4,5). This discordance between LDL-C and atherogenic particle burden has important clinical implications: a substantial proportion of South Asian patients present with ASCVD at LDL-C concentrations below 100 mg/dL (2.6 mmol/L), a threshold at which standard risk calculators would otherwise offer reassurance (1,2).

**Table 1:** Typical lipid phenotype in South Asian versus European-ancestry populations.


LIPID PARAMETER	TYPICAL SOUTH ASIAN PHENOTYPE	TYPICAL EUROPEAN-ANCESTRY PHENOTYPE	CLINICAL IMPLICATION

**LDL-C**	Often similar despite higher ASCVD risk	Variable	LDL-C alone may underestimate risk

**ApoB**	Disproportionately elevated relative to LDL-C	More closely aligned with LDL-C	Greater atherogenic particle burden

**Triglycerides**	Elevated	Lower	Reflects insulin resistance and remnant lipoprotein excess

**HDL-C**	Lower	Higher	Associated with impaired reverse cholesterol transport

**sdLDL proportion**	Increased	Lower	Greater arterial-wall penetrance and oxidisability

**Lp(a) >50 mg/dL (125 nmol/L)**	Frequently elevated	Variable	Independent ASCVD risk; not statin-responsive

**Non-HDL-C**	Often elevated relative to LDL-C	More concordant with LDL-C	Practical ApoB surrogate on a standard panel


Data synthesised from Refs (1,5,25,26). ApoB = apolipoprotein B; HDL-C = high-density lipoprotein cholesterol; LDL-C = low-density lipoprotein cholesterol; Lp(a) = lipoprotein(a); sdLDL = small dense LDL.

The discordance between LDL-C and true atherogenic particle burden is explained by ApoB, which represents one particle per molecule regardless of cholesterol content. ApoB and the ApoB/ApoA-1 ratio have demonstrated stronger associations with ASCVD risk than LDL-C in several South Asian cohorts (5,12,13,14), yet routine ApoB reporting and population-specific decision thresholds remain absent from most clinical guidelines and healthcare systems (5,15). Non-HDL-C, calculable from a standard fasting lipid panel, provides a practical surrogate where ApoB is unavailable (16). The discordance between LDL-C and atherogenic particle burden in South Asians is illustrated in Figure 1.

**Figure 1:**
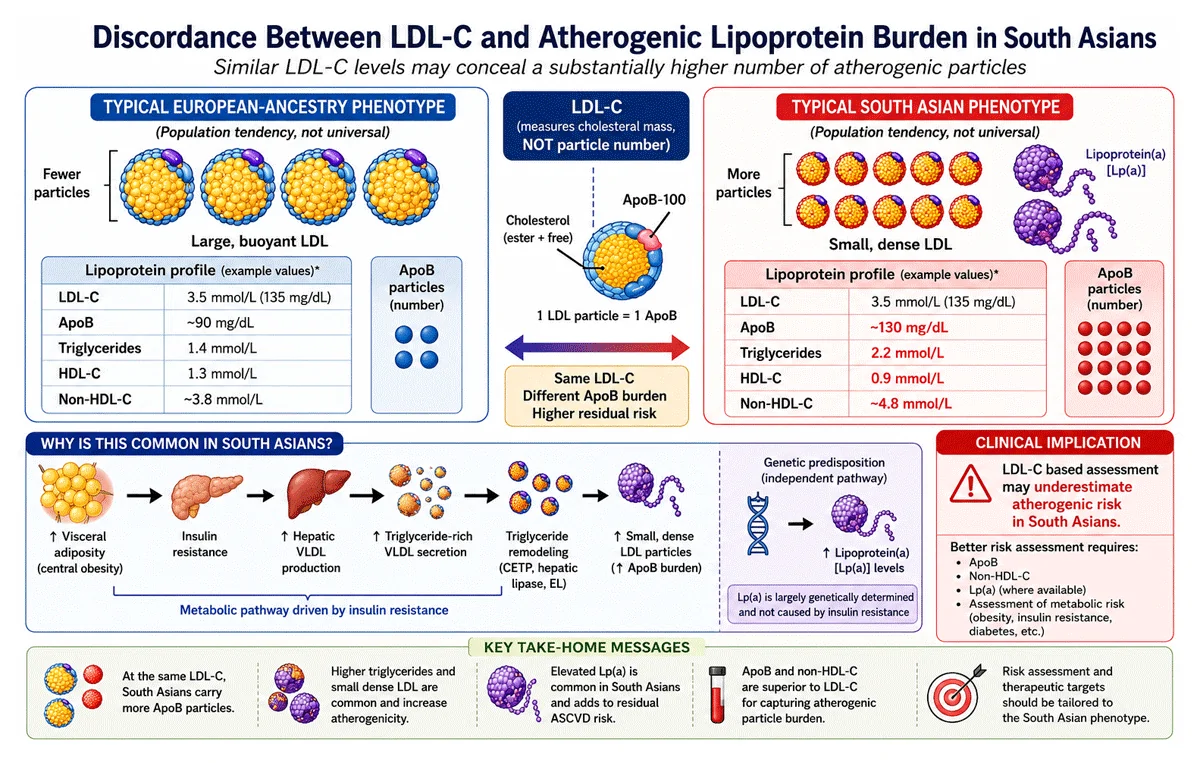
The South Asian lipid paradox. Two patients with identical LDL-C (135 mg/dL, 3.5 mmol/L) have substantially different atherogenic particle burdens. The South Asian phenotype is characterised by a predominance of sdLDL particles and elevated Lp(a), reflected in a higher ApoB concentration despite equivalent LDL-C. Standard cardiovascular risk calculators anchored to LDL-C systematically underestimate risk in this context. ApoB = apolipoprotein B; HDL-C = high-density lipoprotein cholesterol; LDL-C = low-density lipoprotein cholesterol; Lp(a) = lipoprotein(a); TG = triglycerides.

### 2.2 Lipoprotein(a) and residual risk

Lp(a) warrants particular emphasis. A substantial proportion of South Asians, often approaching one quarter in some cohorts, have Lp(a) concentrations exceeding 50 mg/dL (125 nmol/L), conferring independent and additive cardiovascular risk beyond LDL-C (17,18). Data from the Mediators of Atherosclerosis in South Asians Living in America (MASALA) cohort and the UK Biobank demonstrate associations with aortic valve calcification and a steeper risk gradient than observed in European comparators at equivalent concentrations (19). Population-specific reference ranges have not been established, no harmonised screening pathway exists across major guideline frameworks, and South Asian representation in late-phase Lp(a)-targeted therapy trials (pelacarsen, olpasiran, lepodisiran) remains minimal (20). Measurement of Lp(a) at least once in adulthood is supported by the LAI 2023 Consensus Statement IV for risk refinement in Indian patients and by the National Lipid Association (NLA) clinical perspective, and it is now recommended as a Class I indication for all adults in the 2026 ACC/AHA/Multisociety Dyslipidemia Guideline (1,6,10).

### 2.3 Heterogeneity within South Asian populations

The term ‘South Asian’ encompasses populations with diverse genetic ancestry, dietary patterns, and cardiovascular risk trajectories (1,5,10). Despite this diversity, Indian, Pakistani, Bangladeshi, Sri Lankan, and Nepali populations are commonly analysed as a single ethnic category in epidemiological studies and guideline frameworks (1,10). Evidence from South Asian and diasporic cohorts demonstrate variation in lipid profiles and cardiometabolic risk according to sex, urbanisation, and ancestral origin, suggesting that a uniform approach to risk assessment may overlook clinically relevant subgroup differences (21,22). The under-representation of Bhutanese, Maldivian, and Afghan populations in contemporary lipid research further limits understanding of intra-regional variation and remains an important knowledge gap (5).

This heterogeneity has practical implications for clinicians navigating the absence of population-specific risk equations. The existing evidence base is disproportionately Indian and Indian-diasporic in origin, drawn heavily from cohorts such as MASALA (13) and the INTERHEART case-control study (12,14) and consolidated in India-specific frameworks such as the LAI Consensus Statement (10); dedicated evidence for Pakistani, Bangladeshi, Sri Lankan, and Nepali populations is comparatively sparse, and virtually absent for Bhutanese, Maldivian, and Afghan populations. Country-of-origin-specific guidance, where it exists, should take precedence over generic ‘South Asian’ extrapolation. Where it does not exist, clinicians should apply the same risk-enhancing assumptions used for the best-studied group as a precautionary default rather than assume equivalence, while remaining alert that unmeasured differences in diet, urbanicity, and migration generation may shift true risk in either direction.

A related limitation concerns the provenance of the evidence itself. Much of the data most frequently cited to characterise the South Asian lipid phenotype and its cardiovascular consequences, including the MASALA cohort and UK Biobank (19), derives from diaspora populations resident in the US and the UK rather than from South Asians living in South Asia. Diaspora and native populations differ systematically in dietary patterns, environmental exposures, healthcare access, and socioeconomic profile, and diaspora cohorts are additionally subject to a ‘healthy migrant’ selection effect that may not generalise to the broader native population. Because the central argument of this review is precisely the absence of validated risk data in native South Asian populations, caution is warranted in extrapolating diaspora-derived findings, including lipid-phenotype characterisation and Lp(a) prevalence estimates, directly to clinical decision-making in South Asia itself.

### 2.4 Early ASCVD and familial hypercholesterolaemia

The early onset of ASCVD in South Asians, occurring 5–10 years earlier than in European populations, implies that atherogenic dyslipidaemia accumulates during decades that standard screening programmes do not address (2,23). Evidence on screening thresholds in South Asian children and adolescents remains sparse. The co-occurrence of premature coronary artery disease and familial hypercholesterolaemia (FH) in young South Asians is of particular concern, given an estimated burden of approximately five million affected individuals in India alone, the majority undiagnosed, and a mutation spectrum that diverges from Western reference panels (23,24).

## 3. Cardiovascular Risk Stratification in South Asians

### 3.1 Failure of standard risk calculators

Accurate cardiovascular risk stratification is the gateway to appropriate lipid-lowering therapy. Historically, the PCE (ACC/AHA), QRISK3 (NICE), and SCORE2 (ESC/EAS) have been the principal tools used to estimate cardiovascular risk. Developed predominantly in European-origin populations, their performance in South Asians is inconsistent and, in clinically important subgroups, demonstrably inadequate (27,28). Although the PCE has now been replaced by the PREVENT equations in the 2026 ACC/AHA guideline, dedicated validation of PREVENT in South Asian populations is currently lacking (6).

The PCE has been shown both to over- and underestimate 10-year ASCVD risk in South Asian cohorts, with underestimation most pronounced in low- and borderline-risk individuals, precisely the patients in whom a risk-calculator result is most likely to influence the decision to initiate therapy (28). In a large US integrated healthcare cohort, the PCE substantially underestimated absolute 10-year ASCVD risk among South Asians classified as low risk, with observed risk nearly three-fold higher than predicted (4.9% observed versus 1.8% predicted, p < 0.0001); underestimation was more pronounced among those already receiving statin therapy, among whom observed risk exceeded predicted risk by more than five percentage points (e.g., 8.43% observed versus 2.58% predicted) (28). QRISK3 incorporates ethnicity-specific adjustment factors for South Asian groups, although residual miscalibration remains evident in validation studies. In the Southall And Brent REvisited (SABRE) cohort, an earlier ethnicity-stratified validation of QRISK2 found that only around half of observed 10-year cardiovascular risk in South Asian women was captured by the score (predicted-to-observed risk ratio 0.52), compared with closer calibration in European-origin women (ratio 0.73); a gap of similar magnitude was seen with the Framingham score (0.43 versus 0.73) (27). By contrast, an independent validation of QRISK3 in the broader UK Biobank cohort found the score to overpredict risk overall, particularly among older participants, underscoring that population-level and ethnicity-specific calibration are not necessarily aligned and that subgroup-specific validation remains essential (29).

SCORE2 Asia-Pacific has begun recalibration for East and Southeast Asian populations, but India, Pakistan, Bangladesh, Sri Lanka, and Nepal lack contemporary locally validated equations (30,31). Although the 2019 WHO cardiovascular risk charts remain widely used in some South Asian primary-care settings, they are regionally calibrated public-health tools rather than ethnicity-specific lipid-management frameworks and are therefore not discussed further (32).

### 3.2 The 2026 ACC/AHA PREVENT equations and South Asian implications

The 2026 ACC/AHA/Multisociety Dyslipidemia Guideline replaces the PCE with the AHA PREVENT equations for 10- and 30-year risk assessment in adults aged 30–79 years. PREVENT generally generates lower estimated cardiovascular risk than the PCE in derivation and validation cohorts. Whether this improves calibration or instead increases the underestimation of risk among South Asians remains unknown, because dedicated validation studies in South Asian cohorts have not yet been performed (6). Given the consistently lower estimates that PREVENT produces relative to the PCE, prospective validation in South Asian populations is urgently required. Pending such studies, clinicians should apply explicit South Asian risk-enhancer classification under the 2026 framework and maintain a low threshold for coronary artery calcium (CAC) scoring in South Asian patients reclassified downward by PREVENT, relative to their previous PCE estimate. In this context, CAC scoring assumes particular importance as an objective reclassification tool that is independent of risk-equation choice, as discussed below.

### 3.3 Coronary artery calcium scoring

CAC scoring has emerged as the most validated imaging tool for reclassifying South Asians at borderline or intermediate estimated risk. Data from the MASALA cohort demonstrate that CAC scoring meaningfully reclassifies a substantial proportion of South Asian individuals initially categorised as borderline or intermediate risk by conventional calculators. A CAC score of zero identifies a lower-risk subgroup with very low short-term ASCVD event rates, supporting individualised decisions regarding statin initiation (33,34). Guideline uptake of CAC in the South Asian context remains inconsistent across frameworks (Table 2) (8,9,16,35).

**Table 2:** Performance and applicability of cardiovascular risk-stratification tools in South Asian populations.


TOOL	DEVELOPED IN	SA-SPECIFIC ADJUSTMENT	KNOWN LIMITATION IN SOUTH ASIANS	CAC INTEGRATION	LIFETIME RISK

**ATP III (National Cholesterol Education Program Adult Treatment Panel III)**	US	None	No ethnicity modifier; outdated lipid targets	Not included	Not included

**PCE (ACC/AHA 2018)** (8)	US multi-ethnic	SA listed as risk enhancer; no quantitative adjustment	Over- and underestimation reported; underprediction at borderline risk	Class IIa recommendation	Not incorporated

**PREVENT-ASCVD (ACC/AHA 2026)** (6)	US contemporary multi-ethnic cohorts	No quantitative adjustment; SA ancestry treated as a risk-enhancing factor	Not validated in South Asians; generally lower estimates than PCE, risking downward reclassification near thresholds	Recommended for risk refinement when uncertainty persists	10- and 30-year estimates

**QRISK3 (NICE NG238)** (9)	UK population	Includes SA ethnicity within the model	Performance varies by subgroup; NICE advises clinical judgement as risk may be underestimated	Not embedded in pathway	Optional tool for communication; not used to guide statin initiation

**SCORE2 (ESC/EAS)** (16,39)	European	None for South Asia	No validated equation for native SA populations	Class IIa	Not incorporated

**SCORE2 Asia-Pacific** (16,39)	East/SE Asian	Recalibrated for East/SE Asia	Not validated for the SA subcontinent	Not included	Not incorporated

**LAI Consensus IV** (10)	Indian populations	India-specific stratification; supports ApoB, non-HDL-C, Lp(a)	Limited prospective external validation outside India	Considered a useful adjunct	Emphasises early/lifetime risk in younger adults


CAC = coronary artery calcium; PCE = Pooled Cohort Equations; SA = South Asian.

### 3.4 Emerging risk markers

Beyond traditional lipid parameters, several biomarkers may improve cardiovascular risk stratification in South Asians. Lp(a) is elevated in approximately one-quarter of South Asians and functions as an independent ASCVD risk enhancer across contemporary guideline frameworks. Current ACC/AHA, ESC/EAS, and LAI recommendations support at least one lifetime measurement, although no South Asian-specific threshold has been established (5).

ApoB may provide a more accurate estimate of atherogenic particle burden than LDL-C in South Asians, because discordance between LDL-C and ApoB is common in insulin-resistant states characterised by hypertriglyceridaemia and increased concentrations of sdLDL. At any given LDL-C concentration, South Asians frequently exhibit higher ApoB and greater atherogenic particle burden than European populations. Therefore, elevated ApoB despite apparently acceptable LDL-C may identify individuals whose cardiovascular risk is underestimated by conventional assessment (5,17,36).

High-sensitivity C-reactive protein (hsCRP) reflects the systemic inflammation associated with the South Asian metabolic phenotype and is recognised as a risk-enhancing factor in ACC/AHA guidance. Evidence from primary-prevention studies, including the Justification for the Use of Statins in Prevention: an Intervention Trial Evaluating Rosuvastatin (JUPITER) trial, suggests that elevated hsCRP identifies individuals at increased cardiovascular risk despite relatively normal LDL-C and may help refine risk assessment in selected patients (35,37). Polygenic risk scores remain investigational and require validation in large South Asian cohorts before routine clinical implementation. At present, no major framework recommends their routine use in this population (24,38).

### 3.5 The case for lifetime risk

All four contemporary frameworks are anchored to 10-year risk horizons. Given that South Asians experience their first ASCVD event 5–10 years earlier than their European counterparts, this approach systematically deprioritises young South Asian patients whose short-term calculated risk remains low despite a high lifetime atherogenic burden (2). The LAI 2023 Consensus Statement IV explicitly endorses lifetime-risk framing as a supplementary stratification tool in South Asians under 50 years of age, a position now partially addressed by the 2026 ACC/AHA guideline’s incorporation of 30-year risk estimation via PREVENT (6,10). NICE NG238 similarly allows consideration of lifetime risk to support risk communication and lifestyle counselling, although it is not used to determine statin eligibility (9). ESC/EAS has not formally incorporated lifetime-risk estimation into routine treatment algorithms (16,39).

### 3.6 Statin pharmacokinetics and dosing considerations

Even where risk stratification correctly identifies a South Asian patient as requiring statin therapy, none of the frameworks compared in this review address a population-specific consideration in treatment itself. Rosuvastatin plasma exposure (area under the curve) is approximately two-fold higher in Asian subjects than in white subjects of similar age and renal function, independent of body weight, diet, or environment, with Asian Indian subjects specifically shown to have around 60% greater exposure than white subjects in direct comparison (43,44). This elevated exposure is not matched by a proportionally greater LDL-C-lowering effect, so Asian patients achieve comparable percentage reductions in LDL-C at lower doses while experiencing higher systemic drug exposure, and by extension, a plausibly greater risk of dose-dependent myopathy, at doses considered standard in European-origin populations. On this basis, regulatory labelling recommends that Asian patients initiate rosuvastatin at 5 mg once daily rather than the 10–20 mg starting doses used elsewhere (45). A randomised trial conducted specifically in South Asian patients found rosuvastatin to be an effective and generally well-tolerated alternative to atorvastatin in this population, although it was not designed to test dose-adjusted safety outcomes (46). This dosing consideration is one of the few areas in South Asian lipid management where a specific, actionable, and already regulator-endorsed practice change exists today, independent of any future validation of risk equations or treatment targets, yet it is absent from all four major frameworks compared in this review.

## 4. Guideline Comparison and Applicability to South Asian Populations

### 4.1 Points of convergence

Despite differences in architecture and target populations, the contemporary frameworks share several areas of agreement. All endorse high-intensity statin therapy as the first-line treatment in established ASCVD. All recognise South Asian ethnicity as a cardiovascular risk modifier, most explicitly in the 2026 ACC/AHA guideline, which, for the first time, names South Asian and Filipino ancestry as risk-enhancing factors. All support the addition of non-statin agents, principally ezetimibe and PCSK9 inhibitors, when LDL-C targets are not achieved on maximally tolerated statin therapy (6,7,8,9,10,16,39). These areas of convergence provide a clinically stable foundation regardless of which framework a practitioner follows.

### 4.2 Points of divergence

Meaningful divergence exists across five clinically actionable domains, summarised in Table 3.

**Table 3:** Comparison of major lipid guideline frameworks and their applicability to South Asian populations.


DOMAIN	ATP III	ACC/AHA 2018 (7,8)	ACC/AHA 2026 (6)	ESC/EAS 2019 (16)	ESC/EAS 2025 (39)	NICE NG238 (9)	LAI 2023 IV (10)

**Primary treatment target**	LDL-C (numerical)	% LDL-C reduction	Numerical LDL-C and non-HDL-C restored	LDL-C numerical	Unchanged	≥40% non-HDL-C reduction (primary prevention)	LDL-C numerical goals

**Very high-risk LDL-C goal**	<70 mg/dL (<1.8 mmol/L)	≥50% reduction + <70 mg/dL (<1.8 mmol/L) optional	<55 mg/dL (1.4 mmol/L)	<55 mg/dL (<1.4 mmol/L) + ≥50% reduction	Unchanged	Secondary prevention LDL-C ≤77 mg/dL (≤2.0 mmol/L) or non-HDL-C ≤100 mg/dL (≤2.6 mmol/L)	<50 mg/dL (<1.3 mmol/L)

**Extreme-risk LDL-C goal**	Not defined	Not defined	Not defined	<40 mg/dL (<1.0 mmol/L) (IIb)	Added polyvascular disease	Not defined	≤30 mg/dL (≤0.8 mmol/L) (selected)

**High-risk LDL-C goal**	<100 mg/dL (<2.6 mmol/L)	≥50% reduction	≥50% reduction ± <70 mg/dL (<1.8 mmol/L)	<70 mg/dL (<1.8 mmol/L) + ≥50% reduction	Unchanged	Not specifically defined	<70 mg/dL (<1.8 mmol/L)

**Borderline/lower-risk LDL-C goal**	<130 mg/dL (<3.4 mmol/L)	Not specified	<100 mg/dL (<2.6 mmol/L)	Moderate <100 mg/dL (<2.6 mmol/L); low <116 mg/dL (< 3.0 mmol/L)	Unchanged	Focus on >40% non-HDL-C reduction	<100 mg/dL (<2.6 mmol/L)

**Risk calculator**	Framingham	PCE	PREVENT-ASCVD	SCORE	SCORE2 /SCORE2-OP	QRISK3	Indian framework

**SA ethnicity adjustment**	None	Risk enhancer	Risk enhancer	Not named	Explicitly named high-risk modifier	Incorporated in QRISK3	India-focused framework

**Lp(a) screening**	Not included	Optional; once-in-lifetime	Class I once-in-lifetime, all adults	Once-in-lifetime (IIa)	>50 mg/dL (125 nmol/L) risk enhancer (IIa, B)	Not routinely recommended	Recommended in SA adults

**ApoB as target**	Not included	Optional secondary	Reasonable for intensification (ASCVD, diabetes, CKM, hypertriglyceridaemia)	Endorsed secondary	Unchanged	No	Secondary target endorsed

**hsCRP**	Not included	Not specified	Risk enhancer; may guide statin initiation	No formal class	>2 mg/L risk modifier (IIa, B)	Not incorporated	Adjunctive marker

**CAC recommendation**	Not included	Class IIa (intermediate risk)	Risk-refinement pathway when uncertain	Class IIb	Upgraded to IIa, B	Not routinely recommended	Adjunctive refinement

**Non-statin trigger**	LDL-C ≥130 on statin	Goal not met on max statin	Expanded: ezetimibe, PCSK9i, inclisiran, bempedoic acid	Ezetimibe + PCSK9i if goal not met	Added bempedoic acid (I); evinacumab (IIa)	Goal not met in secondary prevention	Goal not met; lower thresholds in very high/extreme risk

**Culturally tailored diet**	None	None	None	None	None	None	Partial; SA diet addressed

**Lifetime-risk framing**	Not incorporated	Not incorporated	30-year estimation incorporated	Not incorporated	Acknowledged as gap	QRISK3 lifetime available	Emphasised, especially younger adults

**Familial hypercholesterolaemia**	Not specified	Not specified	Expanded pathway including genetic testing	Comprehensive; cascade Class I	Added evinacumab HoFH (IIa)	Dedicated referral pathway	Strong emphasis on cascade screening

**South Asian evidence base**	None	None	Limited; PREVENT not validated in SA	None	SA named but not validated	Limited UK SA representation via QRISK3	India-focused; limited external validation


The 2025 ESC/EAS Focused Update names South Asian ethnicity (Box 1) as a high-risk modifier beyond SCORE2/SCORE2-OP, which may support reclassifying an individual to a higher risk category and thereby guide LDL-C goals and intervention thresholds. ApoB = apolipoprotein B; CAC = coronary artery calcium; CKM = cardiovascular-kidney-metabolic; HoFH = homozygous familial hypercholesterolaemia; PCE = Pooled Cohort Equations; PCSK9i = PCSK9 inhibitor; SA = South Asian.

**LDL-C targets**. The most consequential divergence concerns numerical LDL-C goals. ESC/EAS (2019 and 2025 Focused Update) endorses <55 mg/dL (<1.4 mmol/L) for very high-risk patients, with consideration of <40 mg/dL (<1.0 mmol/L) in selected patients at extreme risk (16,39). The LAI 2023 Consensus Statement IV is more intensive still, recommending LDL-C <50 mg/dL (1.3 mmol/L) for very high-risk South Asian individuals and ≤30 mg/dL (≤0.8 mmol/L) for those at extreme risk, reflecting earlier onset and greater ASCVD burden (10). The 2026 ACC/AHA guideline reintroduced numerical goals, recommending <55 mg/dL (<1.4 mmol/L) for very high-risk and <70 mg/dL (<1.8 mmol/L) for high-risk patients, substantially converging with ESC/EAS and LAI (6,8). NICE NG238 differs in retaining a percentage-based primary-prevention target (>40% non-HDL-C reduction) but introducing absolute secondary-prevention targets (LDL-C ≤77 mg/dL (≤2.0 mmol/L) or non-HDL-C ≤100 mg/dL (≤2.6 mmol/L)) in 2023 (9). Consequently, South Asian patients managed in different healthcare systems may receive substantially different treatment goals despite identical risk profiles.

**Operationalisation of South Asian ethnicity**. ACC/AHA identifies South Asian ancestry as a risk-enhancing factor without a quantitative adjustment (7,8,35). ESC/EAS recognises South Asian ethnicity as a risk modifier but does not incorporate it directly into prediction algorithms (16). NICE embeds ethnicity within QRISK3, whereas the LAI framework embeds Indian-population-focused stratification throughout (9,10). As a result, clinicians applying different national guidelines to the same patient may arrive at different risk estimates and treatment thresholds.

**Screening age and Lp(a)**. No consensus exists on the optimal age for lipid screening in otherwise healthy South Asian adults. The LAI and NLA clinical perspectives support earlier assessment because of the high prevalence of premature ASCVD, whereas ACC/AHA, ESC/EAS, and NICE generally follow population-based strategies (1,10). Once-in-a-lifetime Lp(a) measurement is now strongly recommended by ACC/AHA and ESC/EAS and supported by the LAI and NLA, while NICE NG238 remains comparatively less prescriptive (6,9,16,39).

**CAC**. The 2026 ACC/AHA guideline retains CAC as a Class IIa recommendation and adds sex-specific age thresholds (men ≥40 years, women ≥45 years), providing more actionable guidance than the 2018 framework (6). ESC/EAS gives CAC a Class IIa recommendation in its 2025 Focused Update (39). NICE does not embed CAC in the NG238 pathway (9) and the LAI acknowledges it as a potentially useful adjunct without a defined implementation pathway (10). Given the MASALA evidence of reclassification benefit specifically in South Asians, the absence of a harmonised CAC pathway represents a missed opportunity for this population.

**Lifestyle and dietary guidance**. All frameworks endorse a ‘heart-healthy diet’ without addressing the specific dietary exposures prevalent in South Asian populations, including ghee, trans fats, refined grains, high-carbohydrate staples, and vegetarian patterns with suboptimal omega-3 to omega-6 ratios. A more substantive alternative would extend the WHO recommendation to replace saturated and trans fats with unsaturated alternatives (40) into region-specific substitutions: favouring mustard or soybean oil, both comparatively rich in alpha-linolenic acid, over ghee and palm-based cooking fats, shifting the carbohydrate base from refined grains and polished rice towards the whole grains, legumes, and pulses that are already regional staples, and directly addressing the omega-3 deficit common in vegetarian South Asians through plant-based alpha-linolenic acid sources such as flaxseed and walnuts, given limited regional access to marine omega-3. Regional precedents exist on which such guidance could build: the LAI has previously issued consensus dietary guidelines for Asian Indians addressing comparable substitutions (41), and India’s regulatory cap on industrial trans fats at 2% of total fat, recognised by the WHO as a best-practice policy and mirrored by comparable action in Bangladesh, demonstrates that population-level implementation of at least one of these exposures is achievable within the region (42). Extending and harmonising such frameworks across South Asian guideline bodies, rather than defaulting to generic dietary language, would substantially strengthen the practical value of lipid guidance for this population.

### 4.3 Practical implications: which framework to apply?

For clinicians managing South Asian patients, the practical answer is context-dependent (Box 1). In native South Asian settings, LAI 2023 Statement IV provides the most population-appropriate framework and should be the primary reference—though for patients from countries other than India, this remains the best available proxy rather than a validated national framework, consistent with the heterogeneity caveat in Section 2.3. In the UK, NICE NG238 with QRISK3 remains the regulatory standard, but its known limitations in South Asian women and its absence of Lp(a) and CAC pathways should prompt supplementary assessment using LAI or NLA guidance. In the US, the 2026 ACC/AHA framework should be applied, noting three South Asian-specific considerations: South Asian ancestry is now explicitly named as a risk enhancer, PREVENT equations may be used for risk estimation but have not been validated in South Asians and may underestimate risk at borderline and intermediate categories, and Lp(a) measurement is now a Class I recommendation for all patients. In all settings, the more aggressive LDL-C targets of ESC/EAS and LAI are biologically justifiable given the South Asian lipid phenotype, and clinicians should consider adopting them even where national guidance does not mandate them. For resource-limited native South Asian settings, these recommendations are condensed into a stepwise, low-cost-first sequence in Box 2.

**Box 1:** Practical framework selection for lipid risk assessment and management in South Asian populations.


Clinical setting	Primary framework	Additional considerations for South Asian patients

**South Asian countries**	LAI Expert Consensus Statement IV (2023)	Measure ApoB, non-HDL-C, and Lp(a) where available; apply intensive risk-factor modification in high-risk individuals. LAI Statement IV is India-derived; for Pakistani, Bangladeshi, Sri Lankan, and Nepali patients, it is the best available proxy, not a validated national framework, and should be applied with the same caution urged in Section 2.3.

**United Kingdom**	NICE NG238 with QRISK3	QRISK3 incorporates South Asian ethnicity, but risk may still be underestimated in some subgroups. Consider supplementary Lp(a), ApoB, or CAC when treatment decisions remain uncertain.

**United States**	ACC/AHA 2026 Dyslipidemia Guideline	South Asian ancestry explicitly named a risk enhancer. Use PREVENT with awareness of the lack of SA validation—maintain a low threshold for CAC in patients reclassified downward. Once-in-lifetime Lp(a) measurement is now a Class I recommendation for all adults. Numerical LDL-C targets restored: <55 mg/dL (<1.4 mmol/L) very high-risk, <70 mg/dL (<1.8 mmol/L) high-risk.

**Europe (excluding UK)**	ESC/EAS guidelines with SCORE2	Risk tools are not specifically calibrated for South Asians. Additional stratification using ApoB, Lp(a), or CAC may help in selected patients.

**South Asian diaspora**	National guideline of country of residence	Follow local recommendations while recognising the higher cardiometabolic risk profile; incorporate South Asian-specific risk enhancers and targets where appropriate.


ApoB = apolipoprotein B; CAC = coronary artery calcium; LAI = Lipid Association of India; Lp(a) = lipoprotein(a); SA = South Asian.

**Box 2:** A pragmatic point-of-care sequence for lipid management in resource-limited South Asian settings.


The following sequence synthesises the recommendations made throughout this review into an ordered, low-cost-first pathway. It is offered as a starting point for local adaptation and clinical judgement, not as a validated clinical algorithm.

1. Assess conventional risk factors and apply South Asian ethnicity as an automatic risk enhancer, regardless of which calculator is used; treat a ‘low-risk’ result with caution, given the systematic underestimation documented in this review (Section 3.1).

2. Order a standard fasting lipid panel and calculate non-HDL-C at no additional cost; add a one-time Lp(a) and ApoB where accessible (Section 5).

3. Initiate a statin if LDL-C or non-HDL-C exceeds the intensive LAI/ESC-EAS thresholds for the patient’s risk category, even when the calculated 10-year risk is ‘low’, given the underestimation documented in Section 3.1; start rosuvastatin at 5 mg or atorvastatin at 10 mg, consistent with the pharmacokinetic considerations in Section 3.6, rather than at doses standard for European-origin patients.

4. If LDL-C targets are not met on maximally tolerated statin therapy, add ezetimibe as the realistic first-line escalation option; reserve PCSK9 inhibitors, inclisiran, or bempedoic acid for patients who can access them (Section 5).

5. Where risk remains borderline after the above, and CT infrastructure is accessible through tertiary referral, obtain a CAC score to support the decision to intensify or de-escalate therapy (Section 3.3).


## 5. Conclusion: Towards Guideline Convergence

Beyond individual frameworks, this review exposes a structural problem that no single study can resolve. The major guideline frameworks apply different risk tools, treatment targets, screening thresholds, and risk-enhancer algorithms to the same South Asian patient. The result is not clinical flexibility but clinical inconsistency that disproportionately harms a population already at elevated risk. An international consensus process—convened by the LAI, NLA, ESC/EAS, NICE, and the ACC/AHA writing committee, with explicit South Asian representation—to harmonise, at minimum, the risk-enhancer definition, the Lp(a) screening recommendation, and the LDL-C targets for very high-risk South Asian patients, would represent a meaningful and achievable near-term step towards equity.

This harmonisation effort would benefit from a defined mechanism rather than aspiration alone. A practicable model would be a standing South Asian Cardiovascular Risk Working Group, jointly convened by regional professional bodies and international guideline committees (ACC/AHA, ESC/EAS, NICE). Regional representation would be led principally by the LAI together with equivalent cardiology and lipid societies in Pakistan, Bangladesh, Sri Lanka, and Nepal, where these exist or can be newly constituted. Its first task would be a structured consensus process to harmonise risk-enhancer definitions, Lp(a) screening thresholds, and very-high-risk LDL-C targets across frameworks. In parallel, the highest-priority research need is prospective validation: cohort studies based in native South Asian populations, analogous in design to MASALA but conducted in South Asia itself, are needed to generate the outcome data that would convert the biologically plausible recommendations of this review into validated ones, and to test whether tools such as PREVENT and QRISK3 can be recalibrated or replaced with equations specific to South Asian populations. Adequacy, by this measure, would mean a validated South Asian-specific, or, at minimum, major-subgroup-specific risk equation with demonstrated calibration, harmonised risk-enhancer and Lp(a) definitions across major frameworks, outcome-trial-validated LDL-C targets and ApoB/Lp(a) thresholds for South Asian patients, and implementation pathways that are explicitly resource-stratified rather than uniform, so that guidance is achievable in the native settings where the burden of disease is greatest, and not only in diasporic health systems with different resource profiles.

Current guidelines acknowledge but remain incompletely calibrated for South Asian cardiovascular risk. The South Asian lipid phenotype is defined not by elevated LDL-C but by a constellation of atherogenic abnormalities, elevated ApoB, sdLDL, hypertriglyceridaemia, reduced HDL-C, and a high prevalence of elevated Lp(a), that existing risk tools, derived predominantly from European-origin populations, were not designed to capture. Until validated South Asian-specific risk equations exist, a resource-stratified approach to implementation is warranted, since recommendations that presuppose diagnostic infrastructure unavailable in the highest-burden native South Asian settings risk widening, rather than narrowing, the disparities this review seeks to address. Non-HDL-C, calculable at no additional cost from a standard fasting lipid panel, should be reported routinely regardless of setting. ApoB and Lp(a) testing are available through private laboratories in some South Asian urban centres at a cost well below that of CAC scoring or generic testing, though systematic data on patient affordability across the region—particularly outside India and Sri Lanka—are lacking, and these tests remain uncovered by public insurances in most settings. Where accessible, they should be prioritised over CAC scoring as the lower-cost option. However, availability through public sector and rural services remain limited and inconsistent across the region.

CAC scoring requires computed tomography infrastructure and specialist interpretation that are not universally available, particularly outside major urban and tertiary centres. It is unlikely to be feasible as a population-level tool in resource-limited native settings and is better reserved for borderline-risk patients already engaged with tertiary care rather than recommended uniformly.

The same resource lens applies to treatment escalation and not only to testing. Ezetimibe is inexpensive and widely available as a generic across South Asian health systems and can be added to statin therapy at negligible incremental cost. PCSK9 inhibitors, inclisiran, and bempedoic acid, by contrast, remain largely inaccessible in public-sector South Asian healthcare because of cost, and listing them as default escalation options without acknowledging this raises the same equity concern already identified for diagnostic testing. Ezetimibe-based intensification should therefore be regarded as the realistic first-line escalation strategy in most native South Asian settings, with costlier non-statin agents reserved for patients able to access them. Clinicians should apply the intensive LDL-C targets of ESC/EAS and LAI given earlier disease onset and greater atherogenic particle burden. We acknowledge an inherent tension in these recommendations. This review has criticised the use of European-derived risk equations and treatment thresholds in South Asian populations on the grounds of unvalidated extrapolation. The same critique applies, in principle, to our own recommendation of ESC/EAS and LAI LDL-C targets and routine ApoB and Lp(a) measurement, since no dedicated cardiovascular outcome trials have tested these specific targets in South Asian populations, and no South Asian-specific decision thresholds for ApoB or Lp(a) have been established. These recommendations rest on biological plausibility, the same atherogenic mechanisms discussed throughout this review, rather than on outcome-trial validation specific to South Asians, and should be regarded as a pragmatic, evidence-informed default pending such validation rather than as a fully validated target. The answer to the question this review poses is therefore qualified—current guidelines are a necessary foundation but, as presently calibrated, are not yet adequate for South Asian patients.

## Author Informations

Kavindya Fernando is a medical doctor and a trainee in Chemical Pathology. Shamila De Silva is a professor in medicine and an honorary consultant physician with expertise in cardiovascular medicine and dyslipidaemia.
